# hnRNPA2B1-Mediated Extracellular Vesicles Sorting of miR-122-5p Potentially Promotes Lung Cancer Progression

**DOI:** 10.3390/ijms222312866

**Published:** 2021-11-28

**Authors:** Chuang Li, Fang Qin, Wei Wang, Yifan Ni, Mingyu Gao, Mingxiong Guo, Guihong Sun

**Affiliations:** 1 School of Basic Medical Sciences, Wuhan University, Wuhan 430071, China; lic93@whu.edu.cn; 2 Hubei Key Laboratory of Cell Homeostasis, College of Life Sciences, Wuhan University, Wuhan 430072, China; qinfang@whu.edu.cn (F.Q.); 2019202040124@whu.edu.cn (W.W.); niyf97@whu.edu.cn (Y.N.); 2020202040127@whu.edu.cn (M.G.); 3 Hubei Provincial Key Laboratory of Allergy and Immunology, Wuhan 430071, China

**Keywords:** extracellular vesicles, miR-122-5p, non-small cell lung cancer, hnRNPA2B1, RNA-binding protein

## Abstract

Extracellular vesicles (EVs) released by tumor cells play important roles on the remodeling of the tumor–stromal environment and on promoting tumor metastasis. Our earlier studies revealed that miR-122-5p, a type of small non-coding RNA, was dysregulated in non-small cell lung cancer (NSCLC) cell-derived EVs. In this study, we found that miR-122-5p was selectively sorted and secreted into lung cancer EVs through binding to RNA-binding protein hnRNPA2B1. In addition, we found that hnRNPA2B1 interacted with miR-122-5p through the EXO-motif. The delivering of lung cancer EVs-miR-122-5p promoted the migration of liver cells, which may play roles in establishing a pre-metastatic micro-environment and hepatic metastasis of lung cancer. Importantly, our findings revealed the molecular mechanism that RNA-binding protein controls the selective sorting of tumor-derived EV miR-122-5p, which potentially promotes lung cancer progression.

## 1. Introduction

Extracellular vesicles (EVs) are double-membrane-bound vesicles shed from the cell membrane or secreted by the cell, with a diameter of 30 to 1000 nm [1]. EVs are mainly composed of micro-vesicles (MVs) and exosomes (Exo). Micro-vesicles are the vesicles that fall off from the cell membrane with a diameter of about 100–1000 nm [2]. In addition, exosomes are originally generated from late endosomes and then fused with multivesicular bodies and released from cell membrane, with a smaller diameter of about 40 to 150 nm [3]. Considerable research has demonstrated that EVs play important roles on cancer genesis, progression and metastasis through transferring cargoes, including proteins, lipids, DNA and RNA molecules, etc., among different cells [2].

RNAs can be sorted into EVs to be protected from RNase degradation, and internalized by neighboring or distant cells, where they subsequently modulate biological processes [4]. Among the many types of RNA molecules carried in EVs, miRNAs have attracted the most attention due to their roles in gene expression regulation. MicroRNAs (miRNAs) are a class of small (~22 nt) noncoding RNAs that play important regulatory roles on various cellular processes at the posttranscriptional gene regulation level via translational inhibition and mRNA destabilization [5].

Lung cancer has been one of the leading causes of cancer-related death worldwide [6]. In China in 2015, it was estimated that 4,292,000 new cancer cases and 2,814,000 cancer deaths occurred, among which there were estimated 733,300 new cases and 610,200 deaths due to lung cancer. Lung cancer has been the most common incident cancer and the leading cause of cancer death in China [7]. In the United States of America, the average five-year survival rate for lung cancer is only 18%, with most lung cancer patients diagnosed at a late stage [8]. Metastasis of lung cancer is not only the malignant sign and characteristic of lung cancer, but also the main cause of treatment failure and patient death. When lung cancer metastasis occurs in late stage, the corresponding benefit of therapeutic treatments is limited.

More and more studies have shown that extracellular vesicles play an important role in the process of lung cancer metastasis. In recent years, many studies have demonstrated that EV miRNAs are closely associated with lung cancer. For example, miR-21, miR-155, miR-200b, and miR-379 are abnormally expressed in lung cancer-derived exosomes, these miRNAs derived from circulating exosomes can serve as potential noninvasive biomarkers for screening and diagnosing lung cancer [9,10]. In addition, it has been reported that hypoxic bone mesenchymal stem cells-derived exosomal miRNAs promote metastasis of lung cancer cells through exosome-mediated transfer of miR-193a-3p, miR-210-3p and miR-5100 by activating STAT3 signaling-induced epithelial–mesenchymal transformation (EMT) [11].

It has been found that the majority of miRNAs are secreted into EVs in a non-selective manner [12], although some specific miRNAs are selectively sorted into EVs [13,14]. Some RNA-binding proteins (RBPs) have been found to play vital roles on the selective sorting of miRNAs into EVs. For example, a recent study has demonstrated that hnRNPA1 plays an important role in specific packaging of miR-196a into CAF-derived exosomes by binding to a specific motif (UAGGUA) existing at the 5′ end of miR-196a, which plays an active role in HNC progression and chemoresistance [15]. Similarly, SYNCRIP protein binds to specific miRNAs enriched in exosomes sharing a common EXO motif (GGCU), which plays a role on the regulation of miRNA localization [16]. A further study indicated that SYNCRIP-mediated selection of the target miRNAs implies both recognition of the EXO sequence by the NURR domain and binding of the RRM domains to 5’ of this motif [17]. Nevertheless, RBPs regulate miRNA sorting into EVs in both sequence-dependent and sequence-independent manners. For instance, Randy Sheckman and his colleague found that YBX1 protein regulated the selective sorting of miRNAs (such as miR-223) and other kinds of small RNA (including tRNA, Y RNAs, and Vault RNAs) into exosomes by interacting with them, while no specific motif was discovered at binding sites [18,19].

Although many researchers have focused on the regulatory roles of EV miRNA in the development of lung cancer, the functions and mechanism of tumor-derived EVs miRNAs on tumor metastasis are still not known. Our previous studies published in 2018 have demonstrated that miR-122-5p, a tumor suppressor, is selectively enriched in the EVs from lung cancer cell lines and plasma of lung cancer patients [20]. However, miR-122-5p was first found to be a highly expressed liver-specific miRNA, which is extremely low in other tissues and tumor. miR-122-5p is involved in physiological and pathological processes of liver, such as hepatocyte development, differentiation, metabolism, HCV replication, and tumor development [21,22]. We wondered why miR-122-5p was highly enriched in lung cancer-derived EVs, whether EV miR-122-5p played roles in the metastasis of lung cancer, and what was the mechanism of miR-122-5p sorting into EVs and designed experiment to explore about it.

Here, we demonstrated that the selective sorting and secretion of tumor suppressor miR-122-5p into lung cancer EVs was regulated by RNA-binding protein hnRNPA2B1. Moreover, the release of miR-122-5p into EVs was consequential for the progression of lung cancer. In addition, our studies also demonstrated that lung cancer cell-derived EVs promoted the migration of liver cells through delivering miR-122-5p, which might play roles in pre-metastasis micro-environment establishment and potentially influence hepatic metastasis of lung cancer.

## 2. Results

### 2.1. miR-122-5p Is Highly Enriched in EVs from Lung Cancer Cells

**Figure 1:**
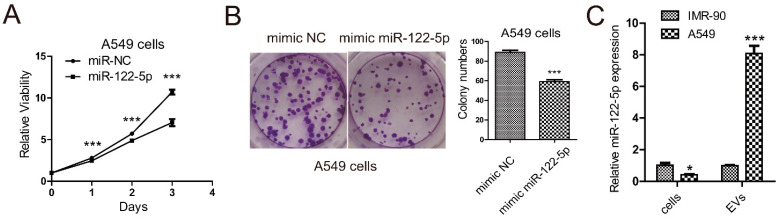
miR-122 is a suppressor of lung cancer A549 cells and is highly enriched in the A549 EVs. (**A**) MTT assay shows the proliferation ability of A549 cells overexpressing miR-122-5p mimic or NC mimic. (**B**) Colony formation assay of A549 cells transfected with constructs expressing miR-122-5p mimic or NC mimic. In addition, the statistical results of colony numbers are shown in the right panel. (**C**) Expression of miR-122-5p in EVs and cell lysates from IMR-90 and A549 cells detected by qPCR. Three independent experiments were performed, and statistical analyses were performed with the Student’s *t* test. *: *p* < 0.05, ***: *p* < 0.001.

Our previous studies published in 2018 demonstrated that the expression of miR-122-5p in plasma EVs from lung cancer patients was extremely high. In addition, we found that expression of miR-122-5p was significantly upregulated in lung cancer cells-derived EVs compared to it in normal lung cell IMR-90 [20]. We wondered whether miR-122-5p was a tumor suppressor for lung cancer cells; thus, we analyzed the influences of miR-122-5p on the viability and proliferation of lung cancer A549 cells. The results of MTT showed that overexpression of miR-122-5p inhibited the viability of A549 cells (Figure 1A), which was in accordance with previous studies. In addition, the colony formation assay also demonstrated that miR-122-5p inhibited the proliferation ability of A549 cells (Figure 1B). We also compared the expression of miR-122-5p in IMR-90 cells and A549 cells and the corresponding levels of miR-122-5p in their EVs (Figure 1C). The results confirmed that miR-122-5p was upregulated in lung cancer EVs, but downregulated in lung cancer cells.

**Figure 2:**
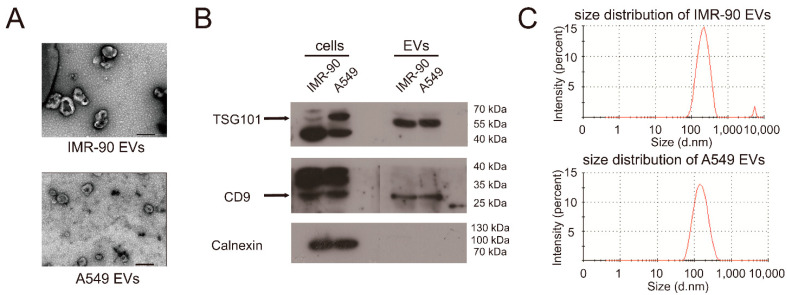
Characterization of EVs secreted by IMR-90 cells and A549 cells. (**A**) Negative staining TEM (×40,000) of EVs from IMR-90 and A549 cells cultured medium. The black solid lines represent 200 nm scale bars. (**B**) Western blot analysis of cell lysate and EVs protein from IMR-90 and A549 cells with antibodies against exosomal markers TSG101 and CD9, and endoplasmic reticulum marker Calnexin, respectively. (**C**) Size distribution of EVs analyzed by nanoparticle tracking analysis.

EVs isolated from cell-conditioned medium after 48 h incubation in EV-free medium through differential ultra-centrifugation (DUC) were subjected to analysis by transmission electron microscopy (TEM), Western blot, and nanoparticle tracking analysis (NTA). Negative staining TEM illustrated that the extracted EVs (pelleting at 100,000× *g*) were mostly in the range of typical exosomal diameter (50–200 nm), and the EVs from IMR-90 were bigger than the EVs from A549 (Figure 2A). We further demonstrated by using Western blot analysis to confirm that the EVs contained the exosomal markers, TSG101 and CD9, but not the intracellular endoplasmic reticulum marker Calnexin (Figure 2B). The above Western blot detections of exosomal markers suggested that the isolated EVs contained abundant exosomes. Besides, the NTA results (Figure 2C, Table 1) showed that the isolated EV fractions were mainly composed of particles in the acceptable size range for EVs from 50 to 400 nm. The NTA results also showed that the two kinds of EVs had similar size distributions, while particle diameters of IMR-90 EVs were slightly bigger than A549 EVs, which was consistent with TEM results (Figure 2A). The NTA results also indicated that the isolated EVs were mainly composed of small size EVs, which indicated that they were exosomes. In addition, overexpression of miR-122-5p in A549 cells also improved its abundance in A549 EVs (Figure S1A,B), suggesting that exogenous miR-122-5p could also be sorted and secreted into EVs. Taken together, the above results indicated that miR-122-5p functioned as a suppressor of lung cancer cells and was selectively sorted and secreted into lung cancer-derived EVs.

### 2.2. RNA-Binding Protein hnRNPA2B1 Interact with miR-122 through EXO-Motif

Next, we wondered how A549 cells specifically sorted tumor-suppressed miR-122-5p into EVs. More and more studies have shown that RNA binding proteins (RBP), such as MVP, SYNCRIP, hnRNPA2B1 and YBX1, are closely related to the process of miRNA sorting and transporting into extracellular vesicles [17,19,23,24]. We wondered if there was also any molecular chaperone similar to RBPs involved in the selective EVs sorting process of miR-122-5p.

**Figure 3:**
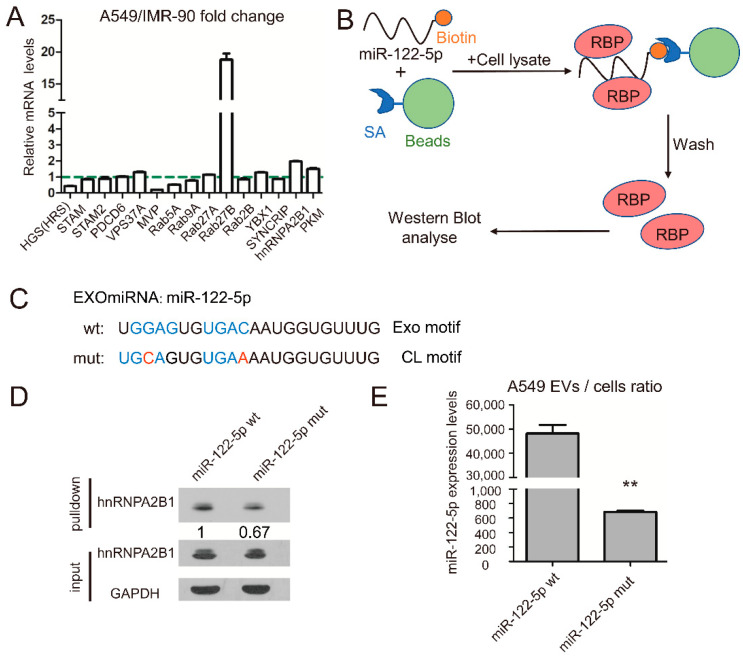
RNA-binding protein hnRNPA2B1 binds miR-122-5p through specific motifs. (**A**) The qPCR results show the mRNA expression levels of 15 candidate EVs sorting-related genes in A549 cells compared to IMR-90 cells. (**B**) Schematic diagram of biotin labelled miRNA-protein pulldown assay. (**C**) Sequences of wild-type miRNAs and their mutated versions used in this study. The mutated miR-122-5p (mut) has two CL motifs, while the wild-type miR-122-5p has two EXO motifs. (**D**) RNA pulldown assay showed that miR-122-5p bound to hnRNPA2B1, and the mutation of EXO motifs on miR-122-5p decreased its binding strength to hnRNPA2B1. (**E**) The mutations changed the ratio of miR-122-5p expression between EVs and cell lysate in A549. Three independent experiments were performed, and the statistical analyses were performed with the Student’s *t* test. **: *p* < 0.01.

If RBPs regulate selective release of miR-122-5p to lung cancer-derived EVs, their abundance may be different in lung cancer and normal lung cells. Therefore, we first detected the expression of 15 candidate RBPs in lung cancer A549 cells and normal lung cells IMR-90 at mRNA level by qPCR, and preliminarily screened out RBPs closely related to lung cancer. Quantitative PCR results showed that hnRNPA2B1 and Rab27b were significantly upregulated in A549 lung cancer cells (Figure 3A). Because miR-122 is downregulated in lung cancer cells and upregulated in EVs derived from lung cancer, we selected hnRNPA2B1 and Rab27b, which were upregulated in lung cancer cells, as the potential research object. Since Rab27b is a protein involved in the releasing of exosomes, the role of Rab27b on the EVs sorting of miR-122-5p may not be specific. In addition, it has been reported that hnRNA2B1 binds miR-198 and miR-601 through specific motif (the Exo-motif), which controls miRNAs sorting into exosomes [24]. Thus, we wondered whether hnRNA2B1 could bind to miR-122 and influencemiR-122 loading into EVs through a similar mechanism.

Hence, the RNA pulldown assay with biotin-labeled miR-122-5p, streptavidin (SA) beads and A549 cell lysate was performed to investigate their interaction (Figure 3B). In miR-122-5p, there are two potential Exo-motifs: typical GGAG and atypical UGAC; thus, we also used biotin-labelled miR-122-5p mutant, which had two CL-motifs rather than Exo-motifs. The RNA pulldown results showed that hnRNPA2B1 bound to miR-122-5p, and the binding ability was inhibited when two EXO-motifs were mutated to CL-motif (Figure 3C,D). In order to investigate whether the EXO-motif impacted miR-122 sorting into EVs, we analyzed the miRNA expression levels in A549 EVs and cell lysates transfected with constructs expressing mutated and wild typed miR-122-5p, respectively. The qRT-PCR results confirmed that EXO-motif mutations inhibited miR-122 loading into A549 EVs (Figure 3E). These results demonstrated that hnRNPA2B1 bound to miR-122-5p through EXO-motif and might influence sorting of miR-122-5p into EVs.

### 2.3. hnRNPA2B1 Regulates the Selective Secretion of miR-122-5p into Lung Cancer EVs

**Figure 4:**
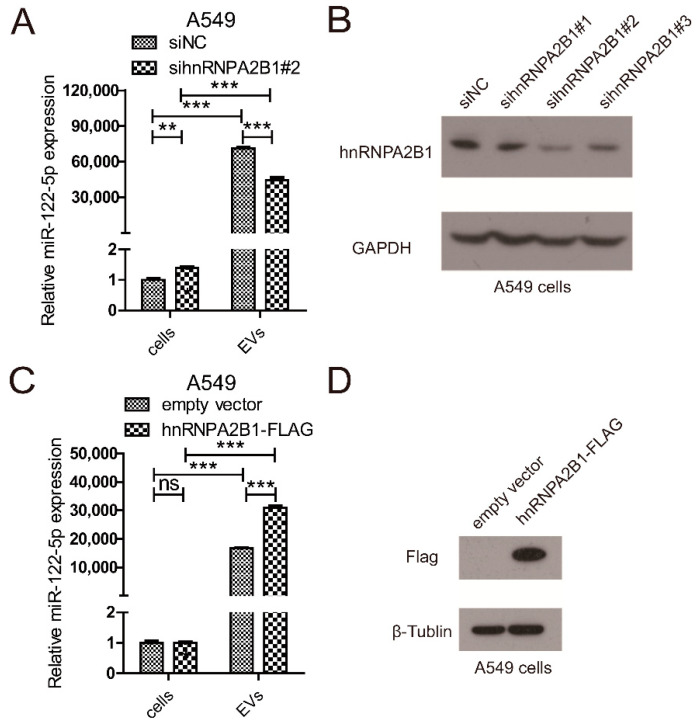
hnRNPA2B1 affects the sorting of miR-122-5p into A549 EVs. (**A**) Expression of miR-122-5p in EVs and cell lysate of A549 transfected with hnRNPA2B1 siRNA or NC siRNA. (**B**) Western blot results show the knockdown efficiency of hnRNPA2B1 siRNA. (**C**) Expression of miR-122-5p in EVs and cell lysate of A549 overexpressed with hnRNPA2B1-flag plasmid or empty vector. (**D**) Western blot results show the overexpression of hnRNPA2B1 using anti-flag antibody. Three independent experiments were performed, and the statistical analyses were performed with the Student’s *t* test. Ns: no significance, **: *p* < 0.01, ***: *p* < 0.001.

Furthermore, we focused on the function of hnRNPA2B1 in regulating the selective release of miR-122 into lung cancer EVs for further research. Loss-of-function assay of hnRNPA2B1 was performed by transfection with siRNA, and the expression of miR-122-5p in cell lysate and EVs was analyzed by qRT-PCR methods (Figure 4A,B). Downregulation of hnRNPA2B1 resulted in significant decrease of miR-122 secretion into A549 EVs after 24h of transfection, while upregulating miR-122 expression in A549 cells. The following gain-of-function assay of hnRNPA2B1 was carried out through transfecting the flag-tagged hnRNPA2B1 overexpression plasmid in A549 cells (Figure 4C,D). We found that overexpression of hnRNPA2B1 significantly increased the miR-122-5p level in A549 EVs after 24h of transfection, while it had no influence on the miR-122-5p level in A549 cells. These results demonstrated that hnRNPA2B1 promoted the selective sorting of miR-122-5p into A549 EVs.

For the reason that Rab27b, which is widely reported to participate in the secretion pathway of exosomes [25], is significantly upregulated in lung cancer cells, we analyzed the influences of Rab27b on the expression of miR-122 in A549 EVs. We found that downregulation of Rab27b significantly reduced the expression of miR-122 in A549 EVs, while the intracellular miR-122 content did not change significantly (Figure S2A,B). These results showed that miR-122-5p was mainly secreted into EVs as exosomes pathway, and Rab27b indirectly affected the secretion of miR-122 by affecting the release of exosomes.

### 2.4. Lung Cancer Cells Secret miR-122-5p-Enriched EVs to Promote Migration of Normal Liver Cells

The miRNA miR-122-5p has been previously reported as liver-specific miRNA, which is mainly expressed in liver tissue. However, our previous published paper indicated that miR-122-5p is abundant in circulating EVs of lung cancer patients. In addition, our analysis of miR-122-5p level also demonstrated the significantly upregulated of miR-122-5p in lung cancer cells-derived EVs. It is widely reported that liver, brain, and bone are the high-risk sites of lung cancer metastasis [26]. More and more evidence has shown that EVs from lung cancer may play an important regulatory role in the distant metastasis of lung cancer [27,28]. However, the function and mechanism of tumor-derived EVs on hepatic metastases of lung cancer is still unknown. Thus, we wondered whether lung cancer A549 cells-secreted miR-122-5p could influence the function of liver cells.

**Figure 5:**
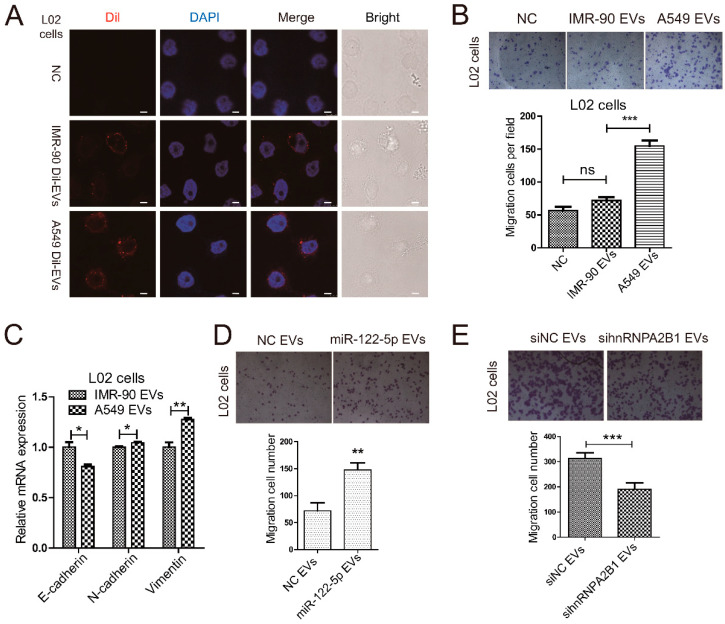
hnRNPA2B1 promotes the migration of L02 cells through regulating the selective secretion of miR-122-5p into A549 EVs. (**A**) Confocal microscopy of L02 cells incubated for 48 h with Dil-labeled IMR-90/A549 EVs (red). The white solid lines represent 5 μm scale bars. (**B**) Representative photographs (100×) of Transwell assay indicated migration of L02 cells treated with EVs from IMR-90 or A549 cells cultured medium. In addition, the statistical results of migrated cells were shown as the down panel. (**C**) qRT-PCR results showed the mRNA expression levels of E-cadherin, N-cadherin and Vimentin in IMR-90 and A549 cells. In addition, the statistical results of migrated cells were shown as the right panel. (**D**) Representative photographs (100×) of Transwell assay showed migration of L02 cells treated with EVs from A549 cells pre-transfected with miR-122-5p mimic or NC mimic. In addition, the statistical results of migrated cells were shown as the down panel. (**E**) Representative photographs (100×) of Transwell assay showed migration of L02 cells treated with EVs from A549 cells pre-transfected with hnRNPA2B1 siRNA or NC siRNA. In addition, the statistical results of migrated cells were shown as the down panel. Three independent experiments were performed, and the statistical analyses were performed with the Student’s *t* test. Ns: no significance, *: *p* < 0.05, **: *p* < 0.01, ***: *p* < 0.001.

In order to investigate the effect of lung cancer-derived EVs on the pre-metastatic microenvironment in liver, we first analyzed whether lung cancer cells-derived EVs could be uptaken by normal liver cells. We isolated EVs from IMR-90 and A549 culture medium by DUC and labeled these EVs with Dil dye. Then, we incubated human normal hepatic cell line L02 cells with Dil-labeled EVs for 48 h. By con-focal microscope (Figure 5A), we found L02 cells can internalize EVs from both IMR-90 and A549 cells. Next, we performed Transwell assay to investigate the influences of lung cancer cells-derived EVs on liver cells migration. The results showed that A549 EVs rather than IMR-90 EVs promoted liver cells (L02 cells) migration (Figure 5B). Epithelial–mesenchymal transformation (EMT) is a pivotal feature of tumor metastasis and may be regulated by EVs’ intercellular transport, so we analyzed the expression of EMT-related markers E-cadherin, N-cadherin, and Vimentin at mRNA levels. The A549 EVs increased the expression of mesenchymal markers N-cadherin and Vimentin, and on the contrary, they decreased the level of epithelial marker E-cadherin compared to IMR-90 EVs (Figure 5C). These qRT-PCR results indicated that A549-derived EVs transmitted more EMT-related properties to normal liver cells, compared to IMR-90 cells.

And we also studied the effect of A549 EVs on viability and proliferation of liver cells using MTT method and colony formation assay. The MTT results showed that the effect of A549-derived EVs on liver cells viability was similar to that of IMR-90-derived EVs (Figure S3A). In addition, colony formation assay also indicated that the influence of A549-derived EVs on the proliferation ability of liver cells had no significant difference compared to that of IMR-90-derived EVs (Figure S3B).

To study the influences of A549 EVs-transferred miR-122-5p on liver cells migration, we overexpressed miR-122-5p in A549 cells and isolated EVs after 24 h culture. Next, L02 cells were treated with the above EVs or negative controls, and migration ability was analyzed by Transwell assay. The results indicated that the increase of miR-122-5p in A549 EVs promoted the migration of L02 cells (Figure 5D). These results showed that lung cancer-derived EVs-miR-122-5p affected the migration of liver cells.

As shown in Figure 4, hnRNPA2B1 bound to miR-122-5p through EXO-motif, which regulated the selective secretion of miR-122-5p into lung cancer EVs. We wondered whether hnRNPA2B1 mediated the function of A549 cells-derived EVs-miR-122-5p on liver cells. Hence, we knocked down the expression of hnRNPA2B1 in A549 cells using siRNA and extracted EVs after 24 h culture. Next, L02 cells were incubated with the above EVs or negative controls, and migration assay was tested by Transwell assay. The Transwell assay results indicated that the downregulation of hnRNPA2B1 in A549 cells promoted the migration of L02 cells (Figure 5E). These results showed that hnRNPA2B1 mediated the selective sorting of mir-122-5p into lung cancer cell-derived EVs, thereby influencing the migration of liver cells.

Collectively, our studies indicated that lung cancer cells-derived EVs were consequential to the migration of liver cells and might play roles on the regulations of EMT process. In addition, our results also suggested that hnRNPA2B1-mediated secretion of EVs-miR-122-5p in lung cancer might participate potentially in the establishment of hepatic pre-metastasis niche.

### 2.5. hnRNPA2B1 Is Closely Associated with the Prognosis of Lung Cancer Patients

**Figure 6:**
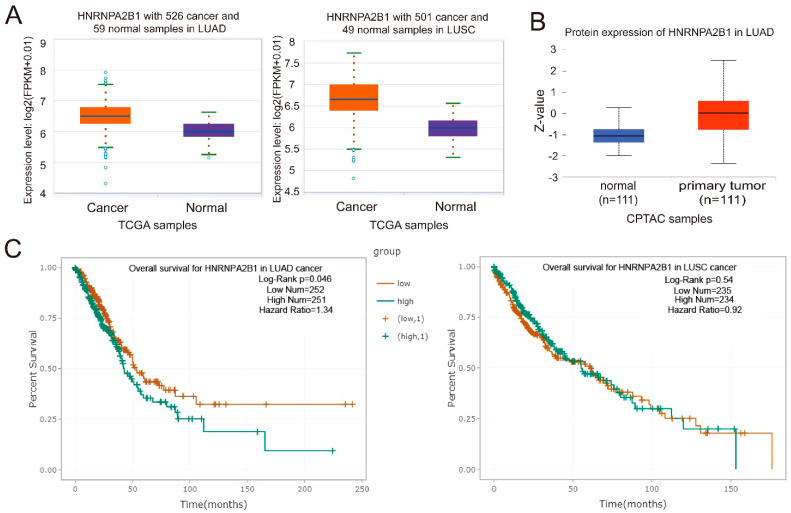
The expression of hnRNPA2B1 is closely related to lung cancer progress. (**A**) The expression levels of hnRNPA2B1 mRNA in LUAD and LUSC patients compared with normal controls (from TCGA database). (**B**) The expression levels of hnRNPA2B1 protein in LUAD patients compared with normal controls (from CPTAC database). (**C**) The overall survival rates of LUAD and LUSC patients with high or low expression levels of hnRNPA2B1 mRNA.

Next, we wondered whether hnRNPA2B1 was closely associated with the genesis and development of lung cancer. With the analysis with clinical data from the TCGA and CPTAC database, we found that hnRNPA2B1 was significantly upregulated in both lung adenocarcinoma (LUAD) and lung squamous cell carcinoma (LUSC) patients at mRNA levels (Figure 6A). The protein expression analysis with clinical data from CPTAC database also indicated that hnRNPA2B1 protein was obviously increased in LUAD patients (Figure 6B), which was in accordance with the mRNA expression data from TCGA. In addition, the analysis with survival data from TCGA database indicated that lung cancer patients with high expression of hnRNPA2B1 had lower overall survival rates (Figure 6C). These analysis with TCGA and CAPTA database showed that hnRNPA2B1 was upregulated in lung cancer patients and was negatively related to the overall survival rates of LUAD and LUSC patients.

Together, our above studies demonstrated that hnRNPA2B1 regulated the selective sorting of miR-122 into EVs through binding with the EXO-motif, and hnRNPA2B1 was tightly associated with lung cancer genesis and development.

## 3. Discussion

In this study, we mainly described the phenomenon that lung cancer cell derived EVs mediated transferring of miR-122-5p which regulated liver cells migration. In addition, we found that the above process was regulated by RNA-binding protein hnRNPA2B1, which bound to miR-122-5p through EXO-motif and regulated its loading into lung cancer EVs.

The EVs mediated cargo delivering from tumor cells to distant organs is not random but selective in some degree. Recent research has shown that integrins on the surface of exosomes are the determinants of tumor cells’ directional metastasis to the target organs. For example, integrins can influence adhesion by remodeling the matrix morphology of cells or organs, integrin αVβ5 are prone to bind specifically to Kupffer cells, and they can promote tumor metastasis to the liver, while integrins α6β4 and α6β1 are prone to bind pulmonary fibroblasts and epithelial cells to migrate to the lung [29]. In this paper, we demonstrated that lung cancer cells-derived EVs could be internalized by the liver epithelial cells. In addition, EVs from lung cancer cells promoted the migration and EMT of liver epithelial cells but had no significant influence on their viability (Figure S3A,B). Our results also demonstrated that tumor-derived EVs produced considerable influence on the pre-metastasis niche formation.

Moreover, it is widely reported that various EV miRNAs play important roles in the genesis and progress of cancer [4]. However, the function of specific miRNA in tumor-derived EV may be different. On the one hand, tumor-derived EVs can transfer oncogenic miRNAs to recipient cells, thereby promote proliferation, migration, extracellular matrix remodeling, tumor colonization and invasion. Oncogenic EV miRNAs promote the formation of a pre-metastatic microenvironment, altering the microenvironment of distant organs to be more suitable for seeding of metastatic cancer cells [30,31]. On the other hand, cancer cell-derived EVs can mediate release of miRNAs as suppressor during tumor progression, which promotes the survival of cancer cells themselves and tumor development [24].

miR-122-5p was first found to be a highly expressed liver-specific miRNA, which involved in many physiological processes such as hepatocyte development, phenotype, differentiation and metabolism, and cell emergency response [32]. Under pathological condition, miR-122-5p promotes the replication of HCV and plays an important role in the genesis and malignant alteration of liver cancer [33,34]. Nevertheless, with the recently rapid development of detection technology, miR-122-5p has also been found its presence in other tissues. In addition, wide range of studies have indicated that miR-122-5p is a tumor suppressor not only in liver cancer but also in other malignant tumors, such as gastric cancer [35] and ovarian cancer [36]. In addition, it has also been reported that miR-122-5p inhibits metastasis and epithelial–mesenchymal transition of non-small-cell lung cancer cells [37].

Our previous study on the small non-coding RNA expression profiles by sequencing with the plasma EVs from NSCLC patients has illustrated that miR-122-5p is downregulated in NSCLC EVs [20]. In addition, we also found that miR-122-5p was highly enriched in EVs from lung cancer cell lines compared to the normal lung cells, while significantly downregulated in lung cancer cells (Figure 1C). Although miR-122-5p is a tumor suppressor, which inhibits the proliferation (Figure 1A,B) and epithelial–mesenchymal transition of NSCLC cells [37], the EVs-packaged miR-122-5p may play the opposite roles on the lung cancer progress. For instance, it has been reported that tumor suppressor miR-193a is selectively sorted into colon cancer exosomes, which paradoxically promotes cancer cell proliferation and tumor progression [23].

For the reason that and miR-122-5p as a hepatic specific miRNA crucial for the physiological and pathological process of liver, the selective enrichment of EV miR-122-5p in lung cancer may influence the tumor metastasis to liver. Hence, we analyzed whether lung cancer cells-derived EV miR-122-5p could influence the hepatic metastasis. The migration assays demonstrated that lung cancer cells-derived EV miR-122-5p promoted the migration of liver cells (Figure 5D). The in vitro data indicated that miR-122-5p-containing EVs from lung cancer might modulate the migratory ability of liver cells in distant microenvironment, which could be beneficial for tumor cells to transfer and penetrate into target organs.

With the rapid advancement of studies on EVs-derived miRNA, more and more researchers are interested in the mechanism how miRNA is sorted into EVs. It has been found that majority of miRNAs are secreted into EVs in a non-selective manner [12], but some specific miRNAs are selectively sorted into EVs [13,14]. The main two routes known for export of miRNA into EVs are Ago-associated pathway and RNA-binding protein-dependent pathway. The RISC (RNA induced silence complex) protein AGO2, regulates the sorting of exosomal miRNAs in a miRNA sequence-independent manner [38]. The RBPs mediate miRNAs’ sorting into EVs in both sequence-dependent and sequence-independent manner. For example, a recent study has demonstrated that hnRNPA1 plays an important role in specific packaging of miR-196a into CAF-derived exosomes through binding to a specific motif (UAGGUA) existing at the 5′ end of miR-196a, which plays an active role in HNC progression and chemoresistance [15]. Similarly, SYNCRIP protein binds specific miRNAs enriched in exosomes sharing a common EXO motif (GGCU), which has a role in the regulation of miRNA localization [16]. Further study indicated that SYNCRIP-mediated selection of the target miRNAs implies both recognition of the EXO sequence by the NURR domain and binding of the RRM domains 5’ to this sequence [17]. Nevertheless, Randy Sheckman and his colleague found that YBX1 protein regulates the selective sorting of miRNAs (such as miR-223) and other kinds of small RNA (including tRNA, Y RNAs, and Vault RNAs) into exosomes by binding with them, and no specific motifs were discovered at binding sites [18,19].

Moreover, hnRNPA2B1 has been found to take part in regulating miRNA, sorting into exosomes in both sequence-dependent and sequence-independent manners. SUMOylated hnRNPA2B1 has been found to bind miR-198 and miR-601 through a common motif (the Exo-motif, G/U-GA-G/C), which promotes sorting of above miRNAs into T lymphocytes-derived exosomes [24]. Conversely, it has been recently reported that hnRNPA2B1 binds miR-503 which has no “Exo-motif”, suggesting that the involvement of hnRNPA2B1 in the export of this microRNA does not follow the same mechanism. In addition, the above paper has shown that binding with hnRNPA2B1 inhibited the exosomal sorting of miR-503, indicating that different ways of interaction with hnRNPA2B1 may influences the sorting and transporting of miRNAs. Our study with RNA pulldown assay has identified that hnRNPA2B1 binds miR-122-5p, and mutations in the two EXO-motifs inhibited the binding effect (Figure 3D). We also found that Exo-motif mutation inhibited the exporting efficiency of miR-122-5p into lung cancer EVs (Figure 3E). However, the exact evidence whether miRNA structure regulates its interaction with hnRNPA2B1 needs further investigation.

Additionally, it has been demonstrated that breast cancer-derived exosomes can transfer miR-122-5p and suppress glucose uptake by niche cells through targeting the glycolytic enzyme pyruvate kinase (PKM), thus increasing nutrient availability for cancer cells in the premetastatic niche and promote tumor metastasis [39]. Hence, NSCLC EVs delivery of miR-122-5p may also function to regulate metabolism, which conversely makes the environment more appropriate for tumor metastasis.

In conclusion, our studies demonstrated that RNA-binding protein hnRNPA2B1-mediated sorting and secretion of miR-122-5p into EVs promoted the progression of lung cancer. In addition, we found that hnRNPA2B1 interacted with miR-122-5p through the EXO-motif. The miR-122-5p-containing lung cancer-derived EVs increased the migration of liver cells, suggesting lung cancer derived EVs might establish pre-metastatic micro-environment promoting the hepatic metastasis of lung cancer. Importantly, this study has established the molecular mechanism that RNA-binding protein could control the selective sorting of miR-122-5p into tumor-derived EV which mediated lung cancer progression.

## 4. Materials and Methods

### 4.1. Cell Culture

Human embryonic lung fibroblast (IMR-90), human lung adenocarcinoma cell line (A549), and human normal hepatocytes (L02) were purchased from the Cell Bank of the Chinese Academy of Sciences (Shanghai, China). IMR-90 were cultured in MEM medium (GIBCO, Grand Island, NY, USA) supplemented with 10% fetal bovine serum (GIBCO), GlutaMAX (GIBCO), MEM non-essential amino acids solution (GIBCO), sodium pyruvate (GIBCO), and penicillin–streptomycin solution. A549 and L02 cells were cultured in DMEM medium (GIBCO) with 10% fetal bovine serum (GIBCO). All cell lines were cultured in a humidified incubator containing 5% CO_2_ at 37 °C.

### 4.2. EVs Isolation

EVs were purified from IMR-90/A549-derived conditioned media by differential ultra-centrifugation. Fetal bovine serum was depleted of EVs by ultracentrifugation at 100,000× *g* overnight at 4 °C, and then the supernatant was sterilized with a 0.45 μm filter (Millipore, Billerica, MA, USA) and stored at −20 °C for later use. When the cell culture density reached 50%, the medium was replaced with EVs-depleted medium. Conditioned media were collected after 48 h and centrifuged at 300× *g* for 10 min, at 2000× *g* for 20 min and at 10,000× *g* for 40 min at 4 °C to remove cells and debris, and EVs were harvested by centrifugation at 100,000× *g* for 90 min (CP80WX, Hitachi, Tokyo, Japan). Next, the pellets were washed with PBS to remove the contaminating protein and then resuspended in PBS.

### 4.3. Transmission Electron Microscopy

For electron microscopy observation, EVs pellets were fixed in 2% paraformaldehyde (PFA), and absorbed onto carbon-coated cooper grids (200 mesh) for 20 min. They were carefully washed with PBS and fix with 1% glutaraldehyde for 5 min. After fixation, samples were washed with PBS and negatively stained with 4% uranyl acetate solution for 5 min. After air drying, sections were observed under TEM (JEM-1400plus, JEOL, Tokyo, Japan).

### 4.4. EVs Size Analysis

To study the size distribution of the purified EVs, Zetasizer (Nano ZS90, Malvern, England, UK) was used, and data analysis was performed with Zetasizer software version 7.13 (Malvern).

### 4.5. Western Blot Analysis

The EV or cell pellets were re-suspended separately in RIPA buffer supplemented with protease inhibitor (TargetMol, Shanghai, China). Twenty micrograms of protein were then separated on a 10% SDS-PAGE gel (Thermo Fisher, Waltham, MA, USA). Proteins were transferred to a polyvinylidene fluoride (PVDF) membrane and blocked with 5% skim milk in Tween/Tris buffered saline (TTBS) for 1h at room temperature. Membranes were then incubated with primary antibodies against TSG101 (ProteinTech, Wuhan, China), CD9 (ProteinTech), GAPDH (Abcam, Cambridge, MA, USA), hnRNPA2B1 (ProteinTech), Rab27b (ProteinTech), Flag tag (Abcam), or Calnexin (ProteinTech) at a dilution of 1:2000 with 3% skim milk diluted in TTBS for 1h at room temperature. After washing several times with TTBS, membranes were incubated with horseradish peroxidase conjugated goat anti-rabbit IgG secondary antibodies (Santa Cruz Biotechnology, Santa Cruz, CA, USA) at a dilution of 1:10,000 in 3% skim milk dissolved in TTBS for 1 h at room temperature. After washing several times with TTBS, membranes were covered with ECL substrate solution and visualized by exposing to film and developing in a film processor.

### 4.6. EVs Uptake Analysis

To observe the uptake of EVs, IMR-90/A549-derived EVs were labeled with the lipophilic dye Dil (Yeasen, Shanghai, China). EVs were suspended in PBS containing 10 μM Dil dye and incubated at 37 °C for 1 h in a rotator under dark conditions. Excess dyes were removed by washed twice by PBS by ultracentrifugation at 100,000× *g*. Then, 100 μg of Dil-labeled EVs were added to L02 cells cultured in confocal dishes, incubated for 24 h. The L02 cells were then washed thoroughly with PBS, fixed with 4% PFA. After treatment with 0.2% Triton-X100, samples were stained with DAPI dye and then observed under a laser confocal microscope (SP8, Leica, Wetzlar, Germany).

### 4.7. Quantitative Real-Time PCR

Total RNA was isolated from cells and EVs using Trizol reagent (Invitrogen, Life Technologies, Carlsbad, CA, USA). Concentration and quality of RNA was measured by Nanodrop2000 (Thermo Fisher). miRNAs were reverse transcribed using the HifairTM II 1st Strand cDNA Synthesis Kit (Yeasen). Quantitative real-time PCR analysis was performed in ABI7500 real-time PCR amplifier (Applied Biosystems, Waltham, MA, USA) using SYBR Green Master Mix (Yeasen). U6 or cel-miR-39 was used as control, and results were analyzed using the 2^−ΔΔct^ calculation method.

### 4.8. Oligonucleotide Transfection

A549 cells were transfected with miRNA mimics or siRNAs at a final concentration of 50 nM using Lipofectamine^®^ 2000 reagent (Invitrogen), following the manufacturer’s instructions.

### 4.9. Cell Migration Assay

The effect of EVs on the migration of L02 was determined by using Transwell Chambers (0.8 μm, 24-well plates, Corning, NY, USA). L02 cells (4 × 10^4^) were suspended in serum-free medium and seeded into the chambers. EVs-depleted medium (500 μL) containing different sources of EVs (100 μg/mL) were added to the bottom. After 24 h co-culture, L02 cells were fixed with 4% paraformaldehyde and stained with 1% crystal violet solution. Samples were observed at microscope to randomly choose six fields for counting the number of migrated cells.

### 4.10. Cell Proliferation Assay

To examine the effects of cell proliferation, MTT and colony formation assays were used. For MTT assay, cells were seeded in four 96-well plates at 1500 cells/well and cultured for 4 days. EVs (100 mg/mL) or equivalent EVs-depleted medium were added. Then 20 μL of MTT solution (Sigma, St. Louis, MO, USA) was added into one of the plates at the same time each day. After continued incubation to culture cells for 4 h, the supernatant was removed and 150 μL DMSO was added into each well. In addition, the absorption value at 570 nm was measured. For colony formation assay, cells were seeded in four 6-well plates at 200 cells/well and cultured for 14 days. EVs (100 mg/mL) or equivalent EVs-depleted medium were added, which was refreshed every 3 days. Cells were fixed with 4% paraformaldehyde and stained with 1% crystal violet solution. Then randomly chose six field for each well were imaged using a digital camera to record the results.

### 4.11. miRNA Mimic and siRNA

miR-122-5p mimics and hnRNPA2B1 siRNA were synthesized by Ribo (Guangzhou, China) and Genepharma (Suzhou, China), respectively. The miR-122-5p mutant sequence of the Exo-motif was UGCAGUGUGAAAAUGGUGUUUG. The miR-122-5p mutant sequence of the hnRNPA2B1 recognition motif was UGGAGUCGGACAAUGGUGUUUG.

### 4.12. miRNA-Protein Pulldown

RNA 3’end desthiobiotinylation kit (Thermo Fisher) was used to attach a single desthiobiotinylated cytidine bisphosphate to the 3’end of the miR-122-5p wild-type (wt) or mutant (mut) strand. Then, the biotinylated RNA was incubated with A549 cell lysates to enrich the proteins that could bind to miR-122-5p wt/mut. Western blot was used to analyze the protein-RNA complexes.

### 4.13. Statistical Analysis

Statistical significance between groups was determined by two-tailed Student’s *t*-tests performed using GraphPad Prism 5.0. Differences were considered significant when *p* < 0.05 (* *p* < 0.05, ** *p* < 0.01, *** *p* < 0.001).

Research manuscripts reporting large datasets that are deposited in a publicly available database should specify where the data have been deposited and provide the relevant accession numbers. If the accession numbers have not yet been obtained at the time of submission, please state that they will be provided during review. They must be provided prior to publication.

## Figures and Tables

**Table 1:** Characterization of EVs particle diameter distribution.

Samples	PDI	Major Peak (nm)	Percentage of Major Peak (%)
IMR-90 EVs	0.384	221.3 ± 6.31	97.9
A549 EVs	0.236	161.5 ± 3.65	100

PDI, polydispersity index.

## Data Availability

The data presented in this study are available within the article text, figures and supplementary materials. The public datasets analyzed during the current study are available in TCGA (https://cancergenome.nih.gov/, accessed on 18 June 2021) and CPTAC (https://proteomics.cancer.gov/programs/cptac, accessed on 18 June 2021).

## Supplementary Materials

The following are available online at www.mdpi.com/article/10.3390/ijms222312866/s1.
