# Supplementary material for: hnRNPA2B1-Mediated Extracellular Vesicles Sorting of miR-122-5p Potentially Promotes Lung Cancer Progression
Source: Int J Mol Sci. 2021 Nov 28;22(23):12866. doi: 10.3390/ijms222312866 (PMC8658035; doi:10.3390/ijms222312866)
Supplement: Supplementary file 1 [file ijms-22-12866-s001.zip › ijms-1465435-supplementary.pdf]

Supplementary figures and legends

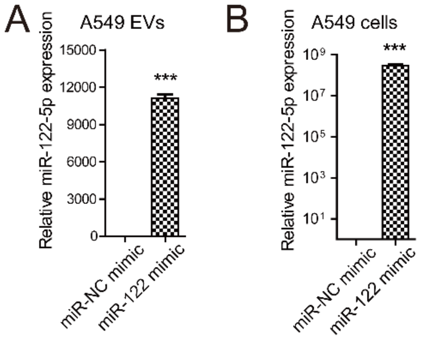

Figure S1. Overexpression of miR-122-5p increases its secretion into EVs. Expression of miR-122-5p in EVs (A) and cell lysate (B) from A549 cells transfected with miR-122-5p mimic or NC mimic. Three independent experiments were performed. Ns: no significance, *: p<0.05, **: p<0.01, ***: p<0.001, compared to the control with the Student's t test.

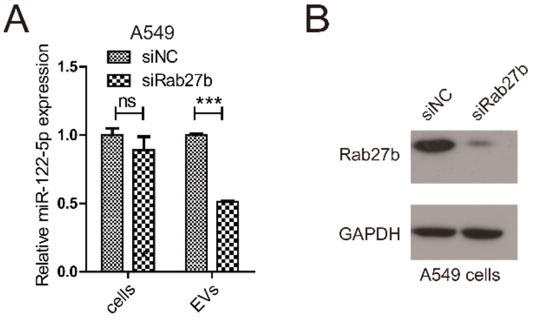

Figure S2. Downregulation of Rab27b inhibits secretion of miR-122-5p into EVs. (A) Expression of miR-122-5p in EVs and cell lysate of A549 transfected with Rab27b siRNA or NC siRNA. (B) Western blot results show the knockdown efficiency of Rab27b siRNA. Three independent experiments were performed. Ns: no significance, *: p<0.05, **: p<0.01, ***: p<0.001, compared to the control with the Student's t test.

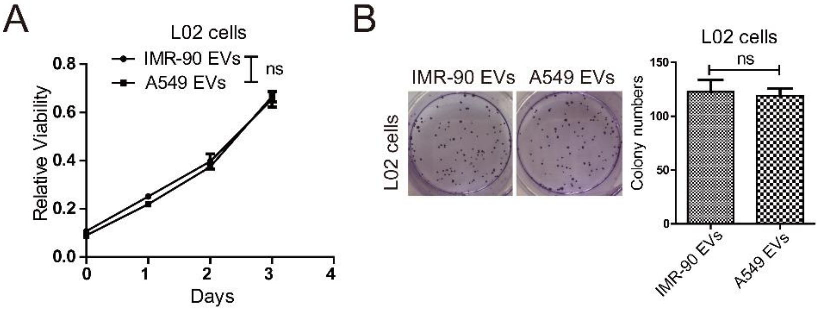

Figure S3. A549 cells derived EVs have no significant influence on the proliferation of L02 cells. (A) MTT assay shows the proliferation ability of L02 cells incubated with EVs from IMR-90 or A549 cells culture medium. (B) Colony formation assay of L02 cells treated with IMR-90 EVs or A549 EVs. And the statistical results of colony numbers were shown as the right panel. Three independent experiments were performed. Ns: no significance, *: p<0.05, **: p<0.01, ***: p<0.001, compared to the control with the Student's t test.
